# Behavioral economic analysis of topiramate pharmacotherapy for alcohol: a placebo-controlled investigation of effects on alcohol reinforcing value and delayed reward discounting

**DOI:** 10.1007/s00213-021-06034-z

**Published:** 2022-01-04

**Authors:** Kimberly Goodyear, Robert Miranda, James MacKillop

**Affiliations:** 1 Center for Alcohol and Addiction Studies, Department of Behavioral and Social Sciences, Brown University, Providence, RI, USA; 2 Center for Alcohol and Addiction Studies, Department of Psychiatry and Human Behavior, Brown University, Providence, RI, USA; 3 Peter Boris Centre for Addictions Research, McMaster University & St. Joseph’s Healthcare Hamilton, Hamilton, ON, Canada; 4 Homewood Research Institute, Guelph, ON, Canada

## Abstract

**Rationale:**

Pharmacotherapies are an important clinical strategy for treating alcohol use disorder and an understanding of their functional mechanisms can inform optimal use. Behavioral economics provides a translational platform that may advance our understanding of the motivational impacts of pharmacotherapies.

**Objectives:**

This secondary analysis study examined the effect of topiramate, a promising pharmacotherapy for treating alcohol use disorder, on two behavioral economic domains, the reinforcing value of alcohol (alcohol demand and alcohol-specific monetary expenditures) and delayed reward discounting (preference for smaller immediate rewards over larger delayed rewards).

**Methods:**

A double-blind randomized placebo-controlled study (*n* = 99) was conducted with non-treatment seeking heavy drinkers, comparing topiramate (target dose of 200 mg/day titrated for 3 weeks and remained at the target dose for 2 weeks) to matched placebo.

**Results:**

We found that compared to placebo, topiramate reduced the reinforcing value of alcohol, as shown by a reduction in two alcohol demand indices (intensity and O_max_), money spent per week on alcohol and an almost a 50% increase in days without expenditures on alcohol from baseline. Directionally consistent patterns were also present for breakpoint and elasticity (*ps* = .08). No significant effects were found for delayed reward discounting.

**Conclusions:**

This study provides evidence that topiramate reduces alcohol’s reinforcing value as measured by alcohol demand and alcohol expenditure. More broadly, these findings support the utility of behavioral economics for understanding how medications reduce alcohol use.

## Introduction

In 2019, more than 6% of individuals ages 18 and older engaged in heavy alcohol use in the past month (SAMHSA, 2020a). Although drinking excessively leads to numerous health problems such as heart disease and cancer (Baan et al., 2007), only about one in 10 individuals with an alcohol use disorder (AUD) receives treatment (SAMHSA, 2020b). Given the high prevalence of heavy drinking, pharmacotherapies have high potential for addressing biological features of AUD, and developing novel efficacious medications is imperative. Currently, disulfiram, acamprosate, and naltrexone are the only medications that are Food and Drug Administration (FDA)-approved for treatment of AUD. Each of these medications has different neuropharmacological mechanisms of action, and empirical support for their efficacy is mixed, with even the most favorable compounds yielding small effect size benefits (Del Re, Maisel, Blodgett, & Finney, 2013; Maisel, Blodgett, Wilbourne, Humphreys, & Finney, 2013).

A promising medication for the treatment of AUD is topiramate (Kenna, 2005; Kranzler & Soyka, 2018), an AMPA/kainite antagonist and facilitator of γ-aminobutyric acid (GABA) receptors. Topiramate reduced drinking in heavy drinkers and individuals with AUD in several randomized clinical trials (Baltieri, Daró, Ribeiro, & de Andrade, 2008; Johnson et al., 2003; Rubio, Martínez-Gras, & Manzanares, 2009), and a meta-analysis found topiramate had significant effects on abstinence, heavy drinking, and craving (Blodgett, Del Re, Maisel, & Finney, 2014). Advancing pharmacotherapy, however, requires not only testing whether medications affect drinking but also how they yield beneficial effects. Indeed, there is considerable interest in understanding the mechanisms of pharmacotherapy effects to optimize clinical use. We previously reported that topiramate reduced alcohol consumption and craving during drinking in heavy drinkers, but craving remained unchanged outside of drinking episodes (Miranda et al., 2008; Miranda et al., 2016).

Behavioral economics examines decision-making processes by combining aspects of psychological and economic science. It is increasingly used to study alcohol and other substance use disorders (Bickel & Marsch, 2001; MacKillop, 2016) as well as the biobehavioral mechanisms by which pharmacotherapies reduce drinking (Bujarski, MacKillop, & Ray, 2012). One important behavioral economics concept is alcohol demand (value of alcohol at escalating costs), which uses demand curve analysis to provide a multifaceted assessment of alcohol reinforcing value. As analogs of progressive-ratio operant schedules, behavioral economic demand tasks can be used in translational medicine development because they putatively parallel operant schedules in animal models (Hursh, Galuska, Winger, & Woods, 2005; Strickland & Lacy, 2020) and can be used to elucidate the pharmacological mechanisms involved when treating disorders. For example, several studies investigating the effects of varenicline on cigarettes smoking found that varenicline reduced maximum financial expenditure (O_max_) (Green & Ray, 2018) and increased sensitivity of consumption to increases in costs (elasticity) for cigarettes (McClure, Vandrey, Johnson, & Stitzer, 2012) compared to placebo. Only one study to our knowledge examined medication effects on alcohol demand. Bujarski et al. (2012) tested the effects of naltrexone on alcohol demand in heavy drinking Asian Americans before and after acute alcohol administration. Naltrexone reduced several alcohol demand indices [intensity (consumption at zero cost), O_max_, and breakpoint (lowest price at which consumption is zero)] compared to placebo. This study provides initial evidence for further examination of the effects of different pharmacotherapies on alcohol demand.

Another important concept of behavioral economics is delayed reward discounting (DRD) (i.e., preference for smaller immediate rewards versus larger delayed rewards). DRD has been demonstrated to be involved with addictive behaviors (Amlung, Vedelago, Acker, Balodis, & MacKillop, 2017; MacKillop et al., 2011), including alcohol (Amlung & MacKillop, 2011) and other substance misuse (Baker, Johnson, & Bickel, 2003; Madden, Bickel, & Jacobs, 1999). Similar to alcohol demand, the relationship between DRD and alcohol motivation/craving has been extensively studied (MacKillop et al., 2010). Therefore, DRD provides another metric for understanding the interplay of pharmacological interventions on alcohol motivation and drinking behaviors. Only one study investigated the relationship of topiramate’s effect on DRD with differential reinforcement for low-rate responding, which provides an efficiency ratio, a measure of impulse control (Rubio et al., 2009), but no effect of topiramate compared to placebo was detected. This measure differs from more ecologically valid measures using financial decision-making, suggesting that further examination is warranted.

In this secondary analysis study, we aimed to expand on the primary aims paper, which investigated the effect of topiramate on drinking, craving, and subjective responses to alcohol use with paired ecological momentary assessment (EMA) and laboratory-based measures (Miranda et al., 2016). The aims of the current study are based on the secondary aims of the primary aims paper, which addresses the limited past research on the relationship between behavioral economics and topiramate by examining alcohol demand with the alcohol purchase task, monetary expenditures measured by the Timeline Followback interview as a novel measure of alcohol-specific and DRD (alcohol and monetary rewards) with the Monetary Choice Questionnaire (MCQ). We hypothesized that topiramate compared to placebo would reduce reinforcing value of alcohol measured with alcohol demand indices and alcohol-specific monetary expenditures and would decrease DRD (i.e., reduce relative immediate reward overvaluation).

## Methods

### Participants

Data for this secondary data analysis are from a larger study that examined the effects of topiramate on drinking, craving and subjective responses to alcohol in non-treatment seeking heavy drinkers in a double-blind randomized placebo-controlled clinical trial (Miranda et al., 2016). The parent study paired ecological momentary assessment (EMA) with laboratory-based alcohol cue reactivity assessment (CRA) methods. Alcohol use was captured with the Timeline Followback (TLFB) interview; craving and subjective responses to alcohol (i.e., stimulation and sedation) were measured via EMA and during the laboratory visits. Participants (*n* = 99) were ≥ 18 years, heavy drinkers (≥14 standard drinks per week in the past 90 days for women and ≥18 drinks for men), and completed the laboratory portion of the parent study (see Table S1 for sociodemographic characteristics and see Miranda et al. (2016) for full exclusion criteria). Participants were randomized to topiramate (*n* = 46) or placebo (*n* = 53) for 5 weeks and those randomized to topiramate reached the target dose of 200 mg/day by week 4 following a 3-week titration period. The study used a per protocol analysis (i.e., only participants who reached the target dose were included in the analysis). The medication capsules contained riboflavin to assess compliance (two participants were excluded for non-compliant urine tests with no riboflavin detected at weeks 4 and 5). Ninety-one participants completed a debriefing questionnaire that asked what condition they were assigned to and 62.5% in the placebo group believed they had a placebo and 86% in the topiramate group believed they had the active drug. Differences across the groups were expected due to the side effects of topiramate. This study was approved by Brown University’s Institutional Review Board (IRB) and informed consent was obtained from all participants.

### Measures

The following measures were administered at baseline and week 4. The alcohol purchase task was used to determine alcohol demand. Participants were asked how many drinks they would consume at escalating amounts of money. The prices included $0.00, $0.01, $0.05, $0.10, $0.50, $1.00, $1.50, $2.00, $2.50, $3.00, $3.50, $4.00, $4.50, $5.00, $6.00, $7.00, $8.00, $9.00, $10.00, $15.00, $20.00, $25.00, $30.00, and $35.00. The TLFB captured daily expenditure on alcohol (Sobell & Sobell, 1992). Three alcohol-specific monetary expenditures were calculated from the TLFB: spent per spending day, spent per week, and percentage of non-spending days. The Monetary Choice Questionnaire (MCQ) is 27-item questionnaire that measured delay discounting at small, medium, and large magnitude rewards (Kirby, Petry, & Bickel, 1999). In addition to domain-general monetary rewards, an alcohol-specific version was also administered, with monetary amounts replaced with standard drinks.

### Data analysis

Data analysis was conducted with the Statistical Package for Social Sciences 26.0 (SPSS 26.0, IBM Corp). To assess any differences between conditions, independent samples *t*-tests and chi-square (χ^2^) analyses were conducted for continuous and binary sociodemographic characteristics. Due to missing data, the sample was reduced for the MCQ (*n* = 93) and TLFB (*n* = 97) analyses. Similar to past work (Murphy & MacKillop, 2006), four behavioral economic indices were calculated from the APT (intensity, O_max_, breakpoint, and elasticity). Intensity (alcohol consumption at zero cost), O_max_ (maximum financial expenditure), and breakpoint (lowest price at which consumption is zero) were calculated based on observed values (Murphy, MacKillop, Skidmore, & Pederson, 2009), either direct participant reports or calculations from raw values. In contrast, elasticity (i.e., sensitivity of consumption to increases in costs) was derived using both mean and individual consumption data in GraphPad Prism (v8, GraphPad Inc.). Nonlinear regression was used to fit and derive parameters from an exponentiated demand equation (Koffarnus, Franck, Stein, & Bickel, 2015): Y = Q_0_ * 10^k(e^−aQ0C^−1)^, where *Y* = quantity consumed at a given price, *Q*_0_ = quantity consumed at zero price, *k* = a constant reflecting the range of consumption values across individuals, *α* = demand elasticity parameter reflecting the rate of consumption decline based on increases in price, and *C* = the cost of the alcohol. The *k* parameter was defined as 4 based on the best fit to the mean baseline demand (*R*^2^ = 0.99). Elasticity for 5 participants could not be modeled due to rectangular demand or too few responses; for the other participants, elasticity was natural log transformed to improve the distribution.

Delay reward discounting for alcohol and money from the MCQ were derived using syntax developed by Gray, Amlung, Palmer, and MacKillop (2016). The *k* value (different from the *k* value above) is a parameter that determines the discounting rate. Participants’ *k* values were based on the rate with the highest consistency across trials and were natural log transformed for analysis. Due to high correlations between *k* values (*p*s < .001), mean values of log-transformed discounting rates were used for small, medium, and large magnitude rewards for both commodities.

To assess the effect of topiramate on behavioral economic alcohol demand, alcohol expenditure, and DRD, 2 × 2 repeated-measures analysis of variance (ANOVAs) were conducted with Condition (topiramate, placebo) as a between-subjects factor and Time (baseline, target dose of 200 mg) as the within-subjects factor. Planned follow-up *t*-tests were conducted for any significant effects. Lastly, to assess associations between behavioral economic alcohol demand, alcohol expenditure, and DRD at the target dose of 200 mg, correlation tests between outcome measures (e.g., alcohol demand indices and alcohol expenditure measures) were employed for the topiramate and placebo groups.

## Results

### Sociodemographic characteristics

**Fig. 1:**
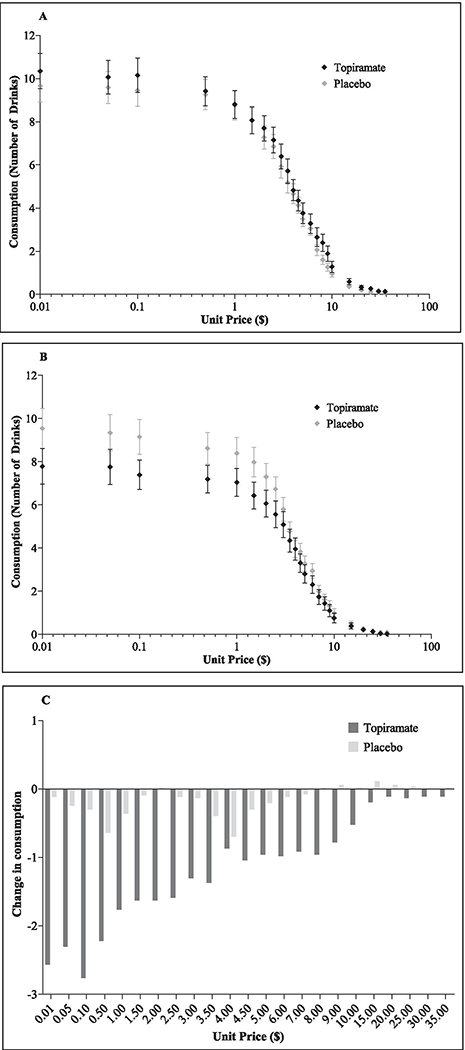
Alcohol demand and expenditure curves. **A** Alcohol demand curves for baseline. **B** Alcohol demand curves at the target dose of 200 mg (and placebo). **C** Differences between the topiramate and placebo conditions from baseline to target dose of 200 mg

No significant differences were found for sociodemographic characteristics between the topiramate and placebo conditions (Table S1). Raw alcohol demand and expenditure values for baseline and target dose of 200 mg showed hypothetical alcohol consumption decreasing as a function of escalating price (Figure 1).

### Alcohol demand indices

**Fig. 2:**
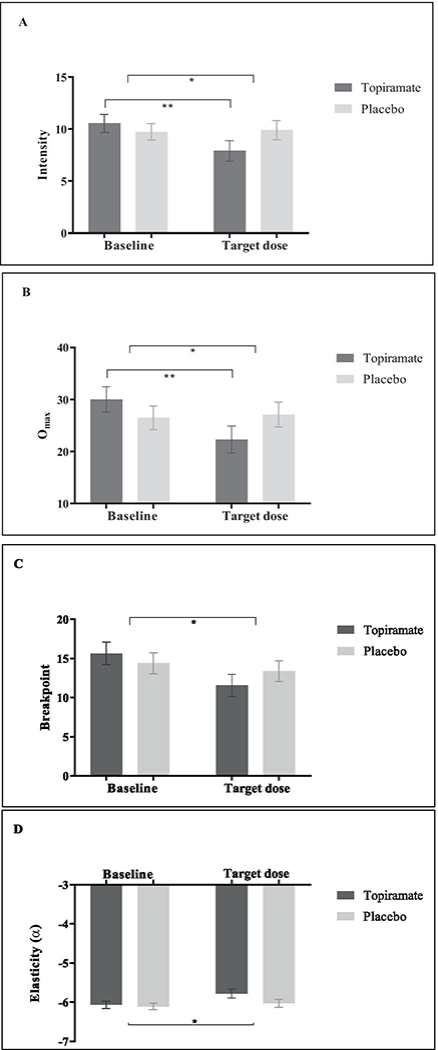
Alcohol demand indices. **A** Intensity (estimated alcohol consumption at zero cost). **B** O_max_ (maximum expenditure for alcohol). **C** Breakpoint (the lowest price at which consumption is suppressed to zero). **D** Elasticity (*α* sensitivity to escalating costs across the demand curve). *p < .05, **p < .001

To assess the effects of topiramate on alcohol reinforcing value, we first investigated alcohol demand and showed a significant main effect of Time for all indices. From baseline to the target dose of 200 mg, alcohol consumption at zero cost (intensity) decreased about 1.1 drinks (*F*(1, 97) = 5.23, *p* = .024, partial *η*^2^ = .051), maximum expenditure for alcohol (O_max_) decreased approximately $3.25 (*F*(1, 97) = 6.97, *p* = .010, partial *η*^2^ = .067), price at zero consumption (breakpoint) decreased about $2.43 overall (*F*(1, 97) = 8.55, *p* < .005, partial *η*^2^ = .081), and sensitivity of consumption to increases in costs (elasticity) increased almost by ½ overall from baseline to target dose of 200 mg (*F*(1, 92) = 9.69, *p* = .002, partial *η*^2^ = .051) (Figure 2). We also demonstrated a Condition × Time interaction effects for intensity (*F*(1, 97) = 6.98, *p* = .010, *η*^2^ = .067) and O_max_ (*F*(1, 97) = 9.72, *p* < .005, partial *η*^2^ = .091). For intensity, follow-up *t*-tests illustrated that alcohol consumption at zero cost decreased about 2.6 drinks for the topiramate group from baseline to target dose of 200 mg (*t*(45) = 3.55, *p* < .001, Cohen’s *d* = −.42) and not for the placebo group (*t*(52) = −0.25, *p* = .804). We found a similar result for O_max_, in which maximum expenditure on alcohol decreased about $7.74 for the topiramate group (*t*(45) = 3.64, *p* < .001, Cohen’s *d* = −.54) and not for the placebo group (*t*(52) = −0.38, *p* = .706). Despite the effect of breakpoint and elasticity not exceeding the significance threshold, we did see a reduction from baseline to target dose of 200 mg in the topiramate group for breakpoint and greater price sensitivity in the topiramate group for elasticity, which is directionally consistent with the our other findings. No other main effects or post hoc tests were found to be significant (all *p*s > .05).

### Alcohol expenditure

**Fig. 3:**
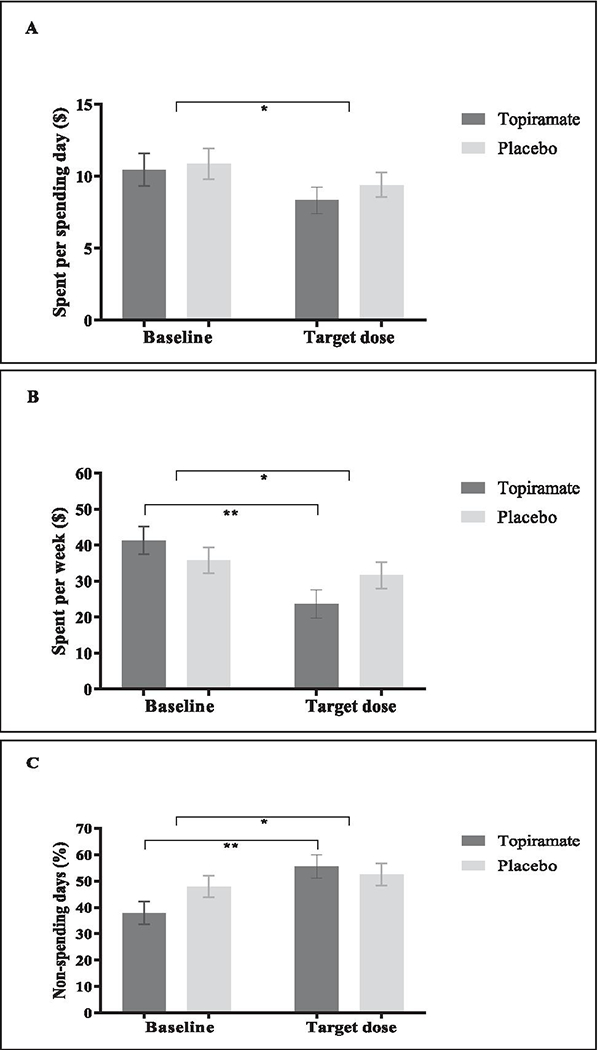
Alcohol expenditure. **A** Amount spent per spending day. **B** Amount spent per week decreased. **C** Percentage of days with no alcohol expenditure. *p < .01, **p < .001

Similar to the alcohol demand indices, we found a significant main effect of Time (baseline to target dose of 200 mg) for alcohol expenditure, indicating that expenditure decreased about $1.75 per spending day (*F*(1, 95) = 7.21, *p* < .01, partial *η*^2^ = .071), decreased $10.43 per week (*F*(1, 95) = 14.27, *p* < .001, partial *η*^2^ = .131) and the percentage of days with no alcohol expenditure (non-spending days) increased about 10.7% (*F*(1, 95) = 17.13, *p* < .001, partial *η*^2^ = .153) (Figure 3). We also found a significant interaction effect of Condition × Time for money spent on alcohol per week (*F*(1, 95) = 5.49, *p* = .021, partial *η*^2^ = .055) and for non-spending days (*F*(1, 95) = 5.75, *p* = .018, partial *η*^2^ = .057). Follow-up *t*-tests showed similar effects from baseline to the target dose of 200 mg, with alcohol expenditure decreasing $17.71 per week for the topiramate group from baseline to target dose of 200 mg (*t*(44) = 5.20, *p* < .001, Cohen’s *d* = .78) and not for the placebo group (*t*(51) = 0.92, *p* = .361) and non-spending days increasing about 17.6% for the topiramate group (*t*(44) = −5.63, *p* < .001, Cohen’s *d* = −.84) and not for the placebo group (*t*(51) = −1.11, *p* = .271). No other main effects or post hoc tests were found to be significant (all *p*s > .05).

### Delayed reward discounting

No significant effects were found for the mean discounting *k* values for the money MCQ or for the alcohol MCQ (all *p*s > .05). Although the effects did not exceed the significance threshold, we saw a trend for the Condition × Time interaction (*F*(1, 91) = 3.34, *p* = .071, partial *η*^2^ = .035) for the money MCQ, indicating increased discounting for the placebo group, but not for the topiramate group.

### Associations between alcohol demand, alcohol expenditure, and delayed reward discounting

When comparing outcome measures in the topiramate group, we found correlations between spent per week and breakpoint (*r*(44) = .31, *p* = .035), alcohol MCQ and O_max_ (*r*(42) = .34, *p* = .023), and money MCQ and O_max_ (*r*(42) = .34, *p* = .026). For the placebo group, we found correlations between spent per spending day and alcohol MCQ (*r*(47) = .34, *p* = .018), spent per spending day and O_max_ (*r*(51) = .44, *p* = .001), spent per spending day and elasticity (*r*(49) = −.30, *p* = .033), spent per week and O_max_ (*r*(51) = .44, *p* = .001), spent per week and elasticity (*r*(49) = −.28, *p* = .045), alcohol MCQ and O_max_ (*r*(47) = .39, *p* = .006), alcohol MCQ and breakpoint (*r*(47) = .31, *p* = .033), and alcohol MCQ and intensity (*r*(47) = .30, *p* = .037).

## Discussion

This study aimed to elucidate the mechanisms of topiramate’s effect on alcohol using behavioral economic indicators of alcohol reinforcing value and DRD. In line with our hypotheses, we first showed that topiramate reduced several alcohol demand indices (intensity and O_max_) and these results are consistent with a previous study that found reduced alcohol demand for participants taking naltrexone (Bujarski et al., 2012). The patterns for breakpoint and elasticity were consistent with these reductions, although they fell short of statistical significance. We also found a more than 40% reduction in money spent on alcohol per week and an almost 50% increase in non-spending days for the topiramate group from baseline to the target dose of 200 mg. These findings demonstrate a real-world application of topiramate’s effects on devaluing the motivation to use alcohol. Other studies have shown that topiramate compared to placebo decreases cocaine use (Johnson et al., 2013) and increases smoking cessation (Oncken et al., 2014), demonstrating a possible non-selective effect of topiramate’s action on blunting responsiveness to rewards. Future studies are needed to understand if the dampened reward response pertains to rewards in general (e.g., food intake) or specific rewards (e.g., drug use).

Typically, drinking behaviors and patterns of use are characterized by actual drinks consumed, but alcohol expenditure provides a different behavioral perspective by showing valuation of a commodity. Our group (Miranda et al., 2016) and others (Baltieri et al., 2008; Johnson et al., 2003) have reported that topiramate reduced drinking and the reduction in money spent per week and the increase non-spending days coincide with those findings. Although the mechanisms of action for naltrexone and topiramate are not the same, the current findings are consistent with topiramate’s devaluation of alcohol, which aligns with the notion that relative drug value is a key mechanisms associated with vulnerability for addiction and recovery (Hogarth & Field, 2020). With the knowledge about the link between behavioral economic demand and alcohol motivation, our behavioral findings can provide further insight into topiramate’s mechanisms.

In terms of DRD, we did not find any significant effects, but showed a trend towards increased discounting rates for the placebo group and not for the topiramate group, as indicated by the money MCQ. We expected that discounting rates for the topiramate group would decrease (preference for larger delayed rewards), but our results fell short of statistical significance. A past study found no differences in the topiramate group for DRD (Rubio et al., 2009) and our study finding also suggests that topiramate did not have an impact on time preferences for rewards, indicating a domain-specific (i.e., alcohol reinforcing value) effect.

Lastly, when comparing associations between alcohol demand, alcohol expenditure, and DRD, we demonstrated medium to moderate correlations between several of the outcome measures in the topiramate and placebo groups. We did find more associations between outcome measures in the placebo group and that was expected due to blunted reward responses that were found in the topiramate group. These findings provide initial evidence that there are associations among the outcome measures, which sets the stage for further investigation on the relationships between these variables.

Our study should be considered in the context of its strengths and limitations. Our sample was limited to only non-treatment seeking heavy drinkers, with a subset diagnosed with AUD. Therefore, the findings may be limited in terms of generalizability; it is important to replicate these results in larger and more representative groups. In addition, the current study was powered to detect medium to large effect sizes, but not small effects. As such, this might have limited our ability to detect significant changes in breakpoint and elasticity. We also could not make direct comparisons to the parent study due to differences in analytical methods (i.e., EMA versus laboratory measures); nonetheless, we found mostly medium effects for our behavioral economic indicators, which are comparable to effects sizes found for other traditional outcomes (e.g., heavy drinking, craving) in topiramate studies (Blodgett et al., 2014). Therefore, there is strong utility in implementing behavioral economic measures in different contexts given the comparable findings to similar clinical endpoints. While the link between hypothetical and actual rewards is well established for alcohol demand and DRD (Amlung, Acker, Stojek, Murphy, & MacKillop, 2012; Amlung & MacKillop, 2015; Lagorio & Madden, 2005), the APT and the MCQ nonetheless used a hypothetical format and participants could have potentially responded differently with actual rewards. On balance, to our knowledge, we are the first to evaluate the mechanisms of topiramate using behavioral economic measures. A double-blind placebo-controlled design provided a rigorous approach to evaluating medication effects. In addition, the study introduced a novel behavioral economic measure of alcohol reinforcing value, estimated by alcohol-specific monetary expenditures on the TFLB, which may have utility in future studies. Lastly, we probed the relationship between outcome measures with correlations due to evidence that cross-sectional mediation can misrepresent causal processes of temporal sequences (Maxwell & Cole, 2007; O’Laughlin, Martin, & Ferrer, 2018); however, subsequent research should expand on our findings by empirically testing the mediating role of alcohol demand and DRD on actual drinking behaviors with longitudinal data.

Collectively, this study expands on past work examining the effect of a pharmacological medications on alcohol demand. We are the first to demonstrate that, compared to placebo, topiramate reduced alcohol’s reinforcing value (intensity and O_max_) and alcohol-specific monetary expenditure. In preclinical models, behavioral choice paradigms have demonstrated that topiramate attenuates alcohol-seeking in rodents (Breslin, Johnson, & Lynch, 2010; Libarino-Santos et al., 2021) and have been implemented to measure reward devaluation in squirrel monkeys through iterative choices of drug and non-drug rewards (Brown et al., 2021). Preclinical drug versus non-drug choice procedures are in line with findings from human pharmacological laboratory studies (Banks & Negus, 2017), and recent literature supports this notion of a translational approach between animal and human studies in regard to behavior economic demand, which provides a segue for understanding mechanisms to lead to pharmacological advances (Smith & Beckmann, 2021; Strickland & Lacy, 2020). While direct pharmacological mechanisms were not measured, we were able to examine topiramate’s behavioral effect on the reinforcing value of alcohol (alcohol demand and alcohol-specific monetary expenditures) and delayed reward discounting. With the limitations of understanding the mechanisms of action for pharmacotherapies in clinical studies, these findings in particular provide an important utility for a translational approach for medication development of pharmacotherapies that affect relative drug value, which can be applicable across different substances. Taken together, there is evidence that topiramate attenuated alcohol reinforcing value, perhaps by way of its inhibition of corticomesolimbic dopamine release. This study provides further insight into the complex behavioral mechanisms involved with topiramate, a promising pharmacological intervention for the treatment of AUD.

## Supplementary Material

1768877_Sup material
