# Supplementary material for: Behavioral economic analysis of topiramate pharmacotherapy for alcohol: a placebo-controlled investigation of effects on alcohol reinforcing value and delayed reward discounting
Source: Psychopharmacology (Berl). Author manuscript; Available in PMC 2023 Jan 4. (PMC8776590; doi:10.1007/s00213-021-06034-z)
Supplement: 1768877_Sup material [file NIHMS1768877-supplement-1768877_Sup_material.docx]

**Table S1.** Sociodemographic characteristics and inferential statistics.

AUD = alcohol use disorder

| Measure | | Topiramate  mean (SD) or N (%) | Placebo mean (SD) or N (%) | Statistic | *p*-value |
| --- | --- | --- | --- | --- | --- |
| Gender | Male | 28 (60.9) | 32 (60.4) | χ^2^ (1) = 0.00 | .960 |
|  | Female | 18 (39.1) | 21 (39.6) |  |  |
| Race/Ethnicity | Caucasian/White | 31 (70.5) | 39 (73.9) | χ^2^ (5) = 3.50 | .624 |
|  | African American/Black | 9 (20.5) | 10 (18.9) |  |  |
|  | Asian | 2 (4.5) | 0 (0.0) |  |  |
|  | Pacific Islander | 0 (0.0) | 1 (1.9) |  |  |
|  | Native American | 1 (2.3) | 1 (1.9) |  |  |
|  | Mixed Race | 1 (2.3) | 2 (3.8) |  |  |
|  | Hispanic/Latino | 4 (8.9) | 7 (13.5) | χ^2^ (1) = 0.50 | .479 |
| Employment | Full Time | 11 (23.9) | 20 (37.7) | χ^2^ (5) = 3.21 | .668 |
|  | Regular Part-Time | 6 (13.0) | 4 (7.5) |  |  |
|  | Irregular Part-Time | 8 (17.4) | 10 (18.9) |  |  |
|  | Summer Only | 2 (4.3) | 2 (3.8) |  |  |
|  | Retired/Disabled | 3 (6.5) | 4 (7.5) |  |  |
|  | Unemployed | 16 (34.8) | 13 (24.5) |  |  |
| Marital Status | Married | 8 (17.4) | 5 (9.4) | χ^2^ (5) = 3.47 | .628 |
|  | Living Together | 5 (10.9) | 10 (18.9) |  |  |
|  | Separated | 3 (6.5) | 2 (3.8) |  |  |
|  | Divorced | 5 (10.9) | 6 (11.3) |  |  |
|  | Widowed | 0 (0.0) | 1 (1.9) |  |  |
|  | Never Married | 25 (54.3) | 29 (54.7) |  |  |
| Income | $0-$9,999 | 14 (31.1) | 14 (26.9 | χ^2^ (13) = 7.74 | .860 |
|  | $10,000 - $19,999 | 7 (15.6) | 11 (21.2) |  |  |
|  | $20,000 - $29,000 | 6 (13.3) | 7 (13.5) |  |  |
|  | $30,000 - $39,000 | 5 (11.1) | 6 (11.5) |  |  |
|  | $40,000 - $49,000 | 3 (6.7) | 4 (7.7) |  |  |
|  | $50,000 - $59,000 | 3 (6.7) | 1 (1.9) |  |  |
|  | $60,000 - $69,000 | 2 (4.4) | 2 (3.8) |  |  |
|  | $70,000 - $79,000 | 0 (0.0) | 2 (3.8) |  |  |
|  | $80,000 - $89,000 | 1 (2.2) | 0 (0.0) |  |  |
|  | $90,000 - $99,000 | 2 (4.4) | 2 (3.8) |  |  |
|  | $100,000 - $109,000 | 0 (0.0) | 1 (1.9) |  |  |
|  | $110,000 - $119,000 | 1 (2.2) | 1 (1.9) |  |  |
|  | $130,000 - $139,000 | 1 (2.2) | 0 (0.0) |  |  |
|  | Greater than $150,000 | 0 (0.0) | 1 (1.9) |  |  |
| AUD | Yes | 23 (50.0) | 24 (45.3) | χ^2^ (1) = 0.22 | .639 |
|  | No | 23 (50.0) | 29 (54.7) |  |  |
| Age |  | 35.91 (12.04) | 35.75 (12.72) | *t*(97) = -0.06 | .950 |
| Education |  | 13.41 (2.08) | 13.77 (2.33) | *t*(97) = 0.81 | .422 |
